# Etiological Development of Alcohol Use and Dependence From Adolescence to Midlife in a Longitudinal Community Study of Twins

**DOI:** 10.1111/acer.70299

**Published:** 2026-04-19

**Authors:** Brooke A. Huizenga, Jordan D. Alexander, Robert F. Krueger, Matt McGue, Sylia Wilson, Stephanie M. Zellers, Scott I. Vrieze

**Affiliations:** ^1^ Department of Psychology University of Minnesota Minneapolis Minnesota USA; ^2^ Institute of Child Development University of Minnesota Minneapolis Minnesota USA; ^3^ Institute for Molecular Medicine Finland University of Helsinki Helsinki Uusimaa Finland

**Keywords:** alcohol consumption, alcohol use disorder, common factor model, genetic, heritability

## Abstract

**Background:**

Alcohol use disorder (AUD) criteria emphasize physiological and psychological consequences of alcohol use without direct evaluation of quantity and frequency of consumption. It remains unclear whether alcohol dependence is an extreme outcome on the alcohol use spectrum or if it represents a separate pattern of behavior with different genetic and environmental influences. To investigate this, we evaluated relationships between alcohol consumption and dependence from adolescence to middle age in a large twin sample.

**Methods:**

Participants were twins (monozygotic pairs = 1205, dizygotic pairs = 676; 52% female) assessed at six waves from adolescence into midlife (age range = 13.6–49.4 years) on frequency of alcohol use, quantity of use, and dependence/abuse symptoms. At each assessment, we fit a longitudinal factor model with a single latent factor loading onto the three alcohol measures. Common and specific factor variance was decomposed into genetic and environmental components.

**Results:**

Alcohol use frequency peaked in early adulthood and remained stable; alcohol use quantity and dependence symptoms peaked in early adulthood and declined thereafter. Factor loadings within waves could not be constrained to be equal at all timepoints without significant detriment to model fit (increases in AIC = 1149 and BIC = 1081). Models constraining loadings at individual timepoints indicate that the measures can be reasonably equated at ages 24 and 29 (decreases in BIC = 3.1 and 2.9, respectively). Genetic factors accounted for about 50% of variance in the latent alcohol behavior factor from ages 14 through 29 but decreased (*p* = 0.007) to 24% by age 37.

**Conclusions:**

Alcohol use and dependence are distinct in adolescence and young adulthood but show convergence in adulthood, such that alcohol dependence symptom count does not clearly measure a construct separate from alcohol consumption longitudinally. These developmental shifts have clinical implications, with AUD screening reliant on assessment of alcohol use patterns expected to perform more reliably in adults than adolescents.

## Graphical abstract

Alcohol consumption and dependence represent distinct constructs during adolescence but converge into a single, unified behavioral pattern in adulthood. Additionally, the genetic influence driving this combined alcohol behavior factor decreases significantly by midlife.

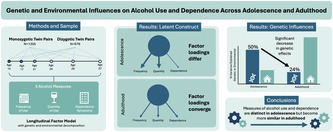

## 1 Introduction

Alcohol use disorder (AUD) as defined by the Diagnostic and Statistical Manual of Mental Disorders (DSM‐5; American Psychiatric Association 2022) is characterized by several psychological and physiological experiences related to the use of alcohol, attempting to measure a style of use that is extensive, habitual, and compulsive, and that negatively affects social or physical functioning. Importantly, the DSM conceptualization of harmful alcohol use has evolved over the past 70 years. Alcoholism in DSM‐II and earlier focuses almost entirely on the extent of alcohol use, including how much and how often a person consumes alcohol. However, starting with the DSM‐III, Alcohol Abuse and Alcohol Dependence diagnostic criteria (now collapsed into a single Alcohol Use Disorder in the DSM‐5) focused on alcohol use that is maladaptive, hazardous, or has negative social, occupational, or physical consequences, without reference to frequency or quantity of use. AUD consists of 11 symptoms, none of which directly measure how much alcohol the individual uses (American Psychiatric Association 2022). Frequency and quantity of use are indirectly measured by symptoms such as tolerance, withdrawal, inability to cut back, and spending a great deal of time on alcohol‐related activities.

Despite not being an explicit diagnostic criterion, measures of use are readily adopted into screening tools for alcohol problems (e.g., alcohol use disorders identification test [AUDIT], NIAAA Single Alcohol Screening Question [SASQ]). These tools have been established as reliable for identifying individuals at risk for having problems related to their alcohol use, in part because they are short and straightforward (Dawson et al. 2005; Hagman 2015; Kim and Hendershot 2020). Explicit relationships between measures of alcohol consumption and standard symptomatology are rarely investigated directly, much less how these relationships may change or unfold across the lifespan, and these relationships may have implications for both theoretical understanding and clinical application and thus should be investigated more clearly.

Past research suggests that excessive consumption of alcohol and AUD are far from perfectly co‐occurring. For example, a large Dutch community study reported that only 17.7% of subjects with AUD reported excessive drinking (i.e., > 14/21 drinks weekly for women/men and at least three 5+ drinking days per week; Tuithof et al. 2014). However, others have found that more severe alcohol problems are significantly related to greater frequency and quantity of use, and individuals who consume alcohol in greater quantities are generally at higher risk for dependence symptoms, though there was significant heterogeneity in these results based on sex and age (Russell et al. 2004). No meta‐analysis has focused on the correlations between use and dependence alone, though a recent meta‐analysis of 612,964 individuals and 10 studies focused on the nexus of use, dependence, and mortality (Carr et al. 2024). Their findings indicated that risk for being diagnosed with AUD increases exponentially with greater use, at least in studies that included a mortality outcome. These results taken together highlight the general positive relationship between alcohol consumption and dependence, though there is conflicting evidence on the strength of the relationship between measures of use and disorder.

Another consideration for the relationship between alcohol use and dependence is that it may change with age. Indeed, use and dependence have clear developmental trajectories. In the United States, alcohol use is generally initiated during adolescence, with peak use during the early 20s and a substantial drop in use thereafter (Richmond‐Rakerd et al. 2017; Zellers et al. 2022). Mean levels of dependence symptoms show a similar pattern, with increases throughout adolescence, a peak in early adulthood, and a decline thereafter (Stephenson et al. 2025; Vrieze et al. 2012). High levels of general and harmful use (i.e., binge drinking, daily drinking) are prevalent among adolescents and young adults (Windle 2003), and these normatively high levels of use may relate to AUD risk in different ways during adolescence compared to adulthood. Longitudinal information on the associations between alcohol use and dependence is key to characterizing any such changes.

Genetic inheritance plays a large role in AUD risk. Twin‐ and family‐based estimates from the United States, Western Europe, and Australia suggest approximately 50% of AUD variance is attributable to additive genetic influences (Verhulst et al. 2015). However, genetic influences are also not static across the lifetime, with evidence showing that the genetic influences via a common factor extracted from drug and alcohol dependence measures become weaker with age (Vrieze et al. 2012). What is more, twin studies routinely report high to very high genetic correlations between use and dependence (*r*_g_ = 0.69–0.91; Dick et al. 2011; Grant et al. 2009; Kendler et al. 2010) and molecular genetic estimates from genome‐wide association studies also find high genetic correlations (0.52–1.0; Kember et al. 2023; Kranzler et al. 2019). However, the heterogeneity of these findings has been the subject of significant scholarship, including a commentary by White and Bierut (2023) comparing and contrasting the many reported results, concluding that the genetic etiologies underlying quantity/frequency of alcohol use, alcohol use problems, and AUD are not identical, but have far more genetic etiology in common than not. Therefore, the genetic overlap between alcohol use and dependence may explain much of their relatedness, but further information is needed to characterize this relationship longitudinally.

The common liability model is a dominant theoretical model that explains the general tendency for the co‐occurrence of substance use and dependence, both within and between substances (Vanyukov et al. 2012). This model posits that a latent trait drives much of the similarity in externalizing disorders, including between alcohol use and dependence. Past research has successfully modeled the co‐occurrence and transmission of externalizing behaviors as a single factor, and highlighted that this latent liability is highly heritable, though substance‐specific etiological influences were non‐negligible (Hicks et al. 2013). Specific influences on different substance behaviors create differences between alcohol use and dependence, and these specific factors themselves are also routinely heritable. One example of a theoretically interesting influence is “addiction resistance,” a concept positing that an individual's discrepancy between reported dependence level and estimated dependence level based on substance use is both heritable and predictable by other health and personality factors (Kendler and Myers 2015). These etiological theories taken together ultimately highlight that alcohol consumption and dependence may be broadly conceptualized as being caused largely by a single latent factor, but the behaviors can be distinct in interesting ways.

Building off this previous literature that has examined both the co‐occurrence of alcohol use and dependence as well as their shared genetic architecture, we add to this literature by characterizing the relationships between these alcohol behaviors and quantifying the genetic and environmental factors that are shared and unique to each measure across major developmental stages. We leverage data from a large community‐based longitudinal twin study dataset with six waves of assessment ranging from adolescence to midlife. Structural equation modeling and a standard biometric decomposition of variance were employed to examine the longitudinal relationship of these measures and quantify the genes and the environmental sources of variation. In line with previous research, we predict that frequency and quantity of alcohol use will be as strongly correlated with dependence as they are to one another, and a common factor extracted from them is highly heritable. However, the magnitude of this similarity as well as how it may change over time remains unclear.

## 2 Materials and Methods

### 2.1 Participants and Procedure

Participants were monozygotic or same‐sex dizygotic twin pairs enrolled through the Minnesota Center for Twin and Family Research (MCTFR) and recruited in three cohorts (MZ twin pairs = 1205, DZ twin pairs = 676; 51.9% female). Twins from two of the three cohorts were representative of nuclear twin families in Minnesota with children born from 1972 to 1984. Slightly over half of the members of an additional cohort (*N*_total_ = 499 twin pairs; birth year range = 1988–1994) were oversampled for elevated disinhibitory behaviors. The current study uses data from six waves of assessment coordinated across the cohorts conducted at target ages of 14, 17, 21, 24, 29 (age SD ≈0.90 at all assessments) and an additional assessment conducted in midlife with a greater age range (mean age = 37 years, SD = 6.54). Further information on these samples can be found in Wilson et al. (2019) and Keyes et al. (2009). Sample descriptive statistics at each wave of assessment are provided in Table 1, with further detail available in the Supporting Information, including the individual cohorts that make up this accelerated cohort design.

**TABLE 1:** Descriptive statistics for overall sample at each wave of assessment.

Wave of assessment	Mean age	(SD)	Total *N*	Complete MZ twin pairs	Complete DZ twin pairs	Female %		Mean	(SD)	Range
Age 14	14.90	(0.55)	2334	729	429	51.4%	Frequency	0.47	(0.97)	[0,5]
							Quantity	0.34	(0.78)	[0,4]
							Dependence	0.08	(0.51)	[0,9]
Age 17	17.85	(0.64)	3485	1094	619	52.2%	Frequency	1.20	(1.08)	[0,5]
							Quantity	1.23	(1.26)	[0,4]
							Dependence	0.51	(1.29)	[0,9]
Age 21	21.08	(0.79)	2711	845	472	55.7%	Frequency	2.11	(1.05)	[0,5]
							Quantity	1.76	(1.01)	[0,6]
							Dependence	0.90	(1.57)	[0,9]
Age 24	24.87	(0.94)	3304	1015	588	53.3%	Frequency	2.41	(1.04)	[0,5]
							Quantity	1.52	(0.86)	[0,6]
							Dependence	0.97	(1.61)	[0,9]
Age 29	29.43	(0.67)	2492	780	427	52.7%	Quantity	1.25	(0.72)	[0,5]
							Dependence	0.60	(1.32)	[0,9]
Age 37	36.85	(6.54)	2211	575	307	57.2%	Frequency	2.37	(1.37)	[0,5]
							Quantity	1.12	(0.77)	[0,6]
							Dependence	0.43	(1.13)	[0,9]

*Note:* Summary statistics for the overall sample, including demographic statistics of participants and central tendency and variability statistics for the measured alcohol variables. Variability in sample size across waves is due to differences in cohort assessment: only two of the three cohorts were fully assessed at waves 14, 21, and 29. For more detail on cohorts included for each wave of assessment, including sample size at each wave by cohort, please refer to the Supporting Information. Information on the scale of Frequency, Quantity, and Dependence variable responses is also in the Supporting Information. Frequency, alcohol use frequency; Quantity, alcohol use quantity; Dependence, DSM‐III‐R Alcohol Abuse and Dependence combined symptom count. Age 29 alcohol frequency missing by design.

### 2.2 Measures

Alcohol consumption was assessed using a modified version of the substance abuse module (SAM; Robins et al. 1987) of the composite international diagnostic interview (Robins et al. 1988) and was defined as average drinking frequency and quantity (per drinking occasion) in the last 12 months. Participant responses were ascertained as ordinal scale categories, inclusive of “0 = Never” to “5 = daily/multiple times per day” for drinking frequency. Drinking quantity was ascertained either continuously or ordinally and harmonized as ordinal scale categories inclusive of “0 = None” to “6 = 30+ drinks” for average drinking quantity per drinking occasion. Both variables were treated as continuous in analyses for computational convenience, and further information on these ordinal scale categories can also be found in the Supporting Information. Alcohol dependence was assessed using the SAM at each of the six waves and defined as the number of symptoms endorsed for both alcohol abuse and alcohol dependence as defined in the DSM‐III‐R, the diagnostic system in place when this longitudinal study began. DSM‐III‐R Alcohol Abuse and Dependence symptoms were combined to best approximate DSM‐5 alcohol use disorder criteria, and we refer to the combined symptom count as “dependence” throughout the results and discussion. For waves at target ages 14 and 17, symptom counts were measured over the past 3 years and lifetime, respectively. However, for the cohort oversampled for disinhibitory behaviors, symptoms were assessed with respect to the past 3 years at both the age 14 and age 17 assessments. At waves 21, 24, and 29, symptom counts were ascertained with respect to the last assessment the individual participated in, usually a 3–5 year recall period. At the age 37 assessment, symptom count was measured with respect to the past year. Past‐year measures of drinking frequency were not administered at the age 29 assessment wave, leaving only quantity and the symptom count at that age. Descriptive statistics for each measure, stratified by zygosity and assessment wave, are shown in Table 1.

### 2.3 Analyses

All analyses were conducted in OpenMx using R version 4.3.0 (Boker et al. 2011; R Core Team 2016). We used biometric multivariate ACE models to decompose the variance–covariance matrix of all measures at all waves into the proportions of the variance attributable to additive genetic (A), shared environmental (C), and nonshared environmental (E) variance components, which represent the proportion of (co)variance in each measure attributable to genetic, shared environmental, and individual‐specific influences. A Cholesky decomposition was used to ensure numerical stability in the solutions. This baseline ACE model of the full variance–covariance matrix imposed no factor structure and constitutes the “fully saturated” estimate of the variance–covariance matrix and means vector in Table 2. All models were estimated using full information maximum likelihood (FIML), and statistical significance was determined by bootstrapping with 1000 replications.

**TABLE 2:** Model fit indices.

Fit indices								
Model	−2 log likelihood	LRT *p*	AIC	BIC	CFI	TLI	Estimated parameters	Degrees of freedom
Fully saturated	114,376.60	—	115,328.55	118,295.32	—	—	476	45,189
Unconstrained	114,930.89	0.000	115,466.89	117,137.26	0.962	0.925	268	45,403
Fully constrained	116,102.13	0.000	116,616.13	118,217.93	0.880	0.772	257	45,414
Age 14 constrained	115,423.65	0.000	115,955.65	117,613.55	0.923	0.851	266	45,405
Age 17 constrained	115,725.12	0.000	116,257.12	117,915.02	0.900	0.806	266	45,405
Age 21 constrained	114,950.49	0.000	115,482.49	117,140.39	0.960	0.923	266	45,405
Age 24 constrained	114,944.24	0.001	115,476.24	117,134.14	0.961	0.924	266	45,405
Age 29 constrained	114,936.25	0.021	115,470.25	117,134.38	0.962	0.925	267	45,404
Age 37 constrained	114,947.57	0.000	115,479.57	117,137.47	0.961	0.923	266	45,405

*Note:* The fully saturated model is a biometric decomposition of the variance–covariance matrix that imposes no factor structure. The unconstrained model imposes the single factor structure shown in Figure 1 but imposes no constraints on factor loadings. The fully constrained model coerces factor loadings within, but not between, waves to be equal at every assessment wave. Age 14–37 constrained models coerce factor loadings within the indicated wave only to be equal. LRT, likelihood ratio test. LRT *p*‐value shown for the unconstrained model indicates the LRT test comparing the unconstrained model to the fully saturated model, and the remaining *p*‐values indicate the LRT tests comparing the constrained models to the unconstrained model. CFI and TLI are also reported, though importantly, the comparative null model for these fit indices included modeling variance for individual variables, as well as covariance for like variables between twins.

**FIGURE 1:**
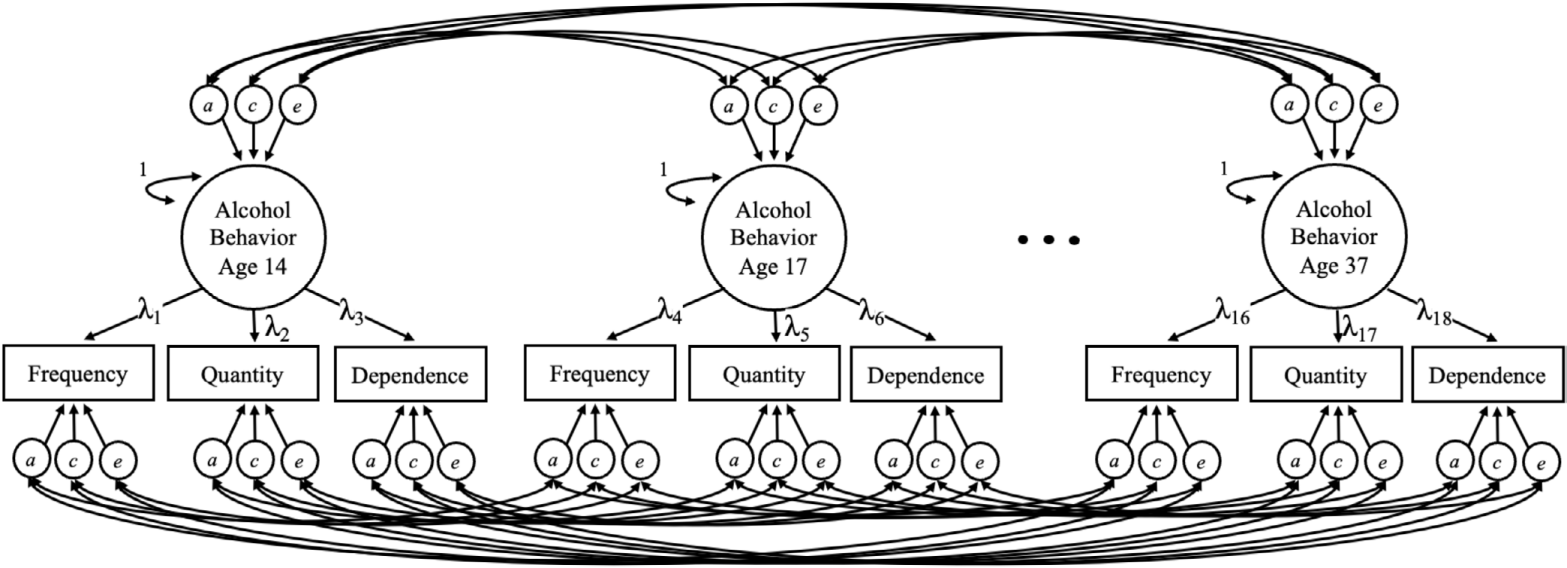
Path diagram of unconstrained common factor model of alcohol use behavior. A single factor model with three specific factors assessing the relationship between measures of alcohol use frequency, alcohol use quantity, and alcohol dependence. Subsequent constrained models coerced factor loadings within waves (e.g., λ_1_ = λ_2_ = λ_3_ and/or λ_4_ = λ_5_ = λ_6_, etc.) to be equal to determine whether specific factors show similar measurement of the latent factor. Alcohol use frequency was not assessed at age 29, and the specific factor was not modeled for that age. Frequency = alcohol use frequency; Quantity = alcohol use quantity; Dependence = DSM‐III‐R Alcohol Abuse and Dependence combined symptom counts; a = variance decomposition attributable to genetic effects; c = variance decomposition attributable to shared environmental effects; e = variance decomposition attributable to nonshared environmental effects.

We next fitted a common pathway model (i.e., a biometrically decomposed confirmatory factor model) that captured loadings of alcohol use frequency, quantity, and alcohol dependence symptom count onto an alcohol behavior latent factor at each of the six waves of assessment. This latent factor represents a common liability to alcohol use and dependence. Both the common and specific factor variances were decomposed into A, C, and E components, as described above. Figure 1 outlines the single factor model. In all models, we controlled for age, sex, and birth year (per McGue and Bouchard 1984). Additionally, alcohol dependence symptom counts at waves 21, 24, and 29 corrected for time since last visit because recall period varied between individuals and waves for these assessments. It was not corrected at ages 14, 17, or 37 because those recall periods were fixed (i.e., either past year, past 3 years, or lifetime). More information on the effect of recall period on alcohol dependence symptom count can be found in the Supporting Information. We fit an “unconstrained” model, which allowed all factor loadings to be freely estimated and compared this to a “fully constrained” model, which required standardized factor loadings for alcohol use frequency, quantity, and dependence to be equal to each other within (but not between) each wave of assessment. Constraining the factor loadings to be equal in the fully constrained model requires observed measures to contribute equally to the latent construct and assumes that they are equally reliable and reflective measures of the latent factor. Therefore, comparing these two models allows us to examine whether these three alcohol measures similarly approximate the same latent construct, and thus act similarly to each other, which would be supported if the fully constrained model did not fit significantly worse than the unconstrained model. This model would require similar measurement of the latent factor by all three alcohol variables at every wave of assessment. In a more nuanced set of analyses, we sought to examine the relationship between the three alcohol variables at individual ages. Here, we then fit additional models where factor loadings at only one wave of assessment per model were constrained to be equal (i.e., the age 14, 17, 21, 24, 29, and 37 constrained models). The best‐fitting single factor model was also compared to a fully saturated model to determine whether a single factor model was appropriate, thereby assessing whether a single underlying construct adequately approximated the observed associations among indicators at an individual age. Model fit was compared using the Akaike information criterion (AIC) and Bayesian information criterion (BIC).

## 3 Results

Table 1 outlines means and standard deviations for symptom counts across waves of assessment. Average alcohol use frequency increased throughout adolescence from 0.47 (~less than 1 drinking occasion per month) at age 14, increasing to 2.41 (~1–3 drinking occasions per month) by age 21, and remaining plateaued at age 37 (= 2.37). Average alcohol use quantity and alcohol dependence symptom count increased into young adulthood (mean quantity at age 14 = 0.34 [less than 1 drink], mean quantity at age 21 = 1.76 [1–3 drinks]; mean dependence at age 14 = 0.08 symptoms, mean dependence at age 24 = 0.97 symptoms) and then declined slightly at the age 29 (mean quantity = 1.25 [1–3 drinks]; mean dependence = 0.60 symptoms) and age 37 assessments (mean quantity = 1.12 [1–3 drinks]; mean dependence = 0.43 symptoms). Correlations among the alcohol use and dependence phenotypes can be found in Table S2.

Overall fits of the unconstrained and fully constrained factor models (i.e., common pathway models) were calculated by comparing them to a fully saturated model, shown in Table 2. There were discrepancies in best fitting model across the different fit indices. According to the AIC, both the unconstrained and fully constrained models fit the data significantly worse than the fully saturated model (AIC increase = 138.34 and 1287.58, respectively); however, the BIC prefers the unconstrained and fully constrained models over the saturated model (BIC decrease = 1161.06 and 77.39, respectively).

Using the unconstrained model as the reference, the fully constrained model fit much worse as shown by an increase in both AIC and BIC (1149.24 and 1080.67, respectively), indicating that factor loadings related to alcohol use frequency, quantity, and alcohol dependence symptom count cannot be coerced to be equal to each other within a given wave at all assessment waves. We also tested each wave individually, constraining the loadings to be equal at that wave and comparing the individually constrained model fits to the fit of the fully unconstrained model. There was no wave where this constraint resulted in better fit according to all fit indices. However, at waves 24 and 29, the BIC preferred the constrained models (decrease of 3.12 and 2.88, respectively), but the AIC did not (increase of 9.35 and 3.36, respectively; full results in Table 2).

**FIGURE 2:**
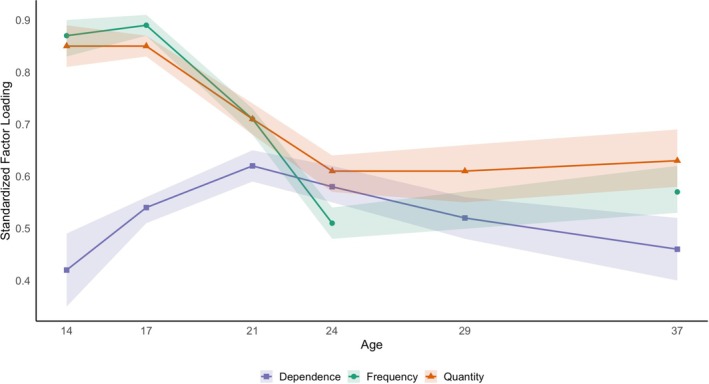
Factor loadings by age. Standardized factor loadings for each of the three manifest variables (frequency, quantity, and dependence) by wave of assessment (i.e., age). Note that alcohol frequency for age 29 is missing by design, and thus there is no factor loading for frequency in the age 29 model. Frequency = alcohol use frequency; Quantity = alcohol use quantity; Dependence = DSM‐III‐R alcohol abuse and dependence combined symptom counts.

Standardized factor loadings for the fully unconstrained model are shown in Figure 2, with point estimates and confidence intervals in Table 3, and a visualization of the factor model with loadings in Figure S1. An examination of the developmental stability in the relationships between the three measures showed that the pattern of factor loadings varied by age of assessment. For assessment waves at ages 14 and 17, standardized factor loadings for both alcohol use frequency (0.87 at age 14 and 0.89 at age 17) and alcohol use quantity (0.85 at age 14 and 0.85 at age 17) were large and comparable to one another. In contrast, standardized factor loadings for alcohol dependence symptoms (0.42 at age 14 and 0.54 at age 17) were moderate and significantly smaller than alcohol use frequency and quantity factor loadings. However, at the other waves, factor loadings for alcohol use frequency, quantity, and alcohol dependence symptom count were either all comparable or factor loadings for alcohol use quantity were more similar to alcohol dependence symptom count than they were to alcohol use frequency. Overall, both the pattern and magnitude of factor loadings for alcohol use frequency, quantity, and alcohol dependence symptom count changed markedly within and across the six waves of assessment, suggesting the measures are operating differently over this 23‐year interval.

**TABLE 3:** Primary results table.

Observed variable/specific factor	Factor Loadings			Biometric Decomposition								
	Latent factor name	Standardized loading		Latent factor			Specific factors					
							A		C		E	
Frequency 14	Alcohol behavior Age 14	0.87	[0.83, 0.90]	A	0.47	[0.21, 0.64]	0.10	[0.02, 0.22]	0.00	[0.00, 0.12]	0.89	[0.74, 0.97]
Quantity 14		0.85	[0.81, 0.89]	C	0.21	[0.07, 0.44]	0.19	[0.02, 0.39]	0.09	[0.00, 0.25]	0.72	[0.55, 0.90]
Dependence 14		0.42	[0.35, 0.49]	E	0.32	[0.25, 0.40]	0.69	[0.36, 0.84]	0.00	[0.00, 0.07]	0.31	[0.16, 0.63]
Frequency 17	Alcohol behavior Age 17	0.89	[0.87, 0.91]	A	0.49	[0.36, 0.62]	0.25	[0.11, 0.34]	0.06	[0.01, 0.17]	0.69	[0.60, 0.80]
Quantity 17		0.85	[0.83, 0.87]	C	0.26	[0.16, 0.38]	0.22	[0.09, 0.31]	0.04	[0.00, 0.12]	0.74	[0.66, 0.84]
Dependence 17		0.54	[0.51, 0.56]	E	0.24	[0.21, 0.28]	0.53	[0.41, 0.60]	0.01	[0.00, 0.09]	0.46	[0.39, 0.53]
Frequency 21	Alcohol behavior Age 21	0.71	[0.68, 0.73]	A	0.55	[0.38, 0.69]	0.09	[0.02, 0.25]	0.23	[0.10, 0.31]	0.68	[0.61, 0.74]
Quantity 21		0.71	[0.68, 0.74]	C	0.18	[0.06, 0.33]	0.05	[0.01, 0.17]	0.08	[0.00, 0.14]	0.87	[0.78, 0.93]
Dependence 21		0.62	[0.59, 0.65]	E	0.26	[0.21, 0.32]	0.28	[0.14, 0.36]	0.02	[0.00, 0.14]	0.70	[0.62, 0.78]
Frequency 24	Alcohol behavior Age 24	0.51	[0.48, 0.54]	A	0.56	[0.40, 0.65]	0.22	[0.10, 0.34]	0.16	[0.06, 0.28]	0.61	[0.56, 0.66]
Quantity 24		0.61	[0.56, 0.64]	C	0.11	[0.04, 0.25]	0.15	[0.03, 0.27]	0.08	[0.01, 0.18]	0.77	[0.69, 0.84]
Dependence 24		0.58	[0.55, 0.62]	E	0.33	[0.26, 0.39]	0.15	[0.09, 0.26]	0.06	[0.00, 0.11]	0.79	[0.72, 0.85]
Frequency 29	Alcohol behavior Age 29	MBD		A	0.54	[0.31, 0.68]	MBD		MBD		MBD	
Quantity 29		0.61	[0.55, 0.66]	C	0.09	[0.02, 0.27]	0.12	[0.02, 0.25]	0.06	[0.00, 0.15]	0.82	[0.72, 0.91]
Dependence 29		0.52	[0.48, 0.56]	E	0.37	[0.25, 0.51]	0.09	[0.05, 0.18]	0.04	[0.00, 0.10]	0.87	[0.79, 0.91]
Frequency 37	Alcohol behavior Midlife	0.57	[0.53, 0.62]	A	0.24	[0.09, 0.39]	0.37	[0.17, 0.47]	0.08	[0.01 0.27]	0.55	[0.48, 0.62]
Quantity 37		0.63	[0.58, 0.69]	C	0.05	[0.01, 0.18]	0.10	[0.03, 0.31]	0.08	[0.01, 0.14]	0.82	[0.66, 0.89]
Dependence 37		0.46	[0.40, 0.52]	E	0.70	[0.56, 0.81]	0.06	[0.02, 0.19]	0.12	[0.02, 0.20]	0.82	[0.72, 0.88]

*Note:* Results shown under Factor Loadings reflect the standardized factor loadings for the unconstrained model relative to the latent factor (“Alcohol Behavior [wave of assessment]”) they load onto. Results shown under *Biometric Decomposition* reflect the estimated proportion of variance attributable to additive genetic effects (A), shared environmental effects (C), and nonshared environmental effects (E) for both the latent factor and specific factors. Values for factor loadings and biometric decomposition are shown as value [95% CI]. Alcohol use frequency at age 29 is missing by design. MBD, missing by design; Frequency, alcohol use frequency; Quantity, alcohol use quantity; Dependence, DSM‐III‐R alcohol abuse and dependence combined symptom count.

We tested the magnitude, stability, and change in genetic and environmental influence of both the common and specific factors between each wave of assessment. The biometric decomposition of the latent and specific factors into the proportion of variance accounted for by genetic, shared environment, and nonshared environmental factors is shown in Table 3. Heritability of the latent factor was moderate and stable from ages 14 to 29 (0.47 at age 14, 0.54 at 19; *p* = 0.90 for the difference in these two quantities) with a decline in heritability at age 37 (0.24; *p* = 0.007 for the decline between ages 29 and 37). The nonshared environment component was also stable from ages 14 to 29 (i.e., 0.32 and 0.37 for ages 14 and 29, respectively; *p* = 0.70) with an increase at age 37 (0.70; *p* = 0.001 for the increase). Shared environmental effects on the latent factor remained stable between ages 14 and 37 (0.21 and 0.05, respectively, *p* = 0.65 for a change). Heritability of the alcohol use frequency and quantity specific factors was stable longitudinally (frequency heritability = 0.10 at age 14 and 0.37 at age 37, *p* = 0.15 for a change; quantity heritability = 0.19 at age 14 and = 0.10 at age 37, *p* = 0.76 for a change). Heritability of the alcohol dependence symptom count specific factor was moderate in adolescence (0.69 at age 14) and decreased in adulthood (0.06 at midlife; *p* = 0.002 for the decrease). Genetic correlations for latent and specific factors are found in Tables S4 and S5, respectively. The proportion of specific factor variance accounted for by shared environment was small or null for all measures and at all waves of assessment (ranged from 0.00 to 0.23), and both the effect of shared and nonshared environment for all three measures remained stable (*p* ranged from 0.28 to 0.95).

## 4 Discussion

The purpose of this study was to understand how measures of alcohol use frequency, alcohol use quantity, and alcohol dependence symptom count relate to each other from adolescence through middle adulthood, and to decompose these relationships into genetic and environmental sources of variation. Factor loadings relating measures of alcohol use frequency, alcohol use quantity, and alcohol dependence symptom count could not be equated at all waves of assessment without significant detriment to model fit, indicating that these three measures do not approximate an alcohol behavior latent construct equally at all ages. Therefore, they do not measure the exact same construct to the same extent over the 23‐year period (on average) represented in this study.

In a more focused analysis of individual ages, it appears that the measures of frequency and quantity of use are more similar to each other than they are with alcohol dependence symptom counts and thus load more heavily onto the latent factor at age 14, 17, and even 21. This pattern subsides at older ages. By age 24, all measures are approximately equally loading onto the latent factor, producing factor loadings of similar magnitude that can be constrained as equal; therefore, all three alcohol measures roughly equally measure the latent alcohol behavior construct at this age, at least according to some, but not all fit indices. The discrepancy in these fit statistic results is due to the fact that BIC, AIC, and likelihood ratio tests have different purposes and qualities, which depend on the unknown true model (Vrieze 2012). It should also be noted that starting at age 24 and onwards when factor loadings become more similar between constructs, loadings for frequency and quantity also decrease in magnitude, indicating that these measures are becoming less correlated with age, such that their measurement of the latent factor approximately equals that of dependence. Nevertheless, these results provide some support for our hypothesis that measures of alcohol use and dependence function similarly at these ages. Therefore, all three measures may be interpreted as equally measuring the latent construct in adulthood and could be used interchangeably to do so. These findings are generally aligned with previous research that has highlighted the general positive relationship between alcohol use and dependence (Carr et al. 2024), though discrepancies between use and dependence at younger ages further highlight that this relationship may vary by age (Russell et al. 2004).

In a closer examination of the factor loading point estimates, they largely remain similar within age for ages 21 and older, indicating that these measures are functioning similarly in adulthood. Though, as mentioned above, they largely remain statistically different. Additionally, the rank order of factor loadings also changes over time, with no clear pattern of which measures relate most to each other longitudinally. As illustrated by their similar factor loadings, frequency and quantity are more related to each other than they are to dependence symptomatology in adolescence, but by the age 37 assessment, alcohol use quantity and alcohol dependence symptom count have more similar factor loadings, indicating they are more related to each other than either is to alcohol use frequency when accounting for variance shared by all three measures. Simple correlations between the three alcohol measures show a similar pattern, whereby the three measures are generally moderately to largely correlated overall, and alcohol use frequency and quantity are usually, but not always, more similar to each other than they are to alcohol dependence (see Table S2). The fact that the patterns of factor loadings change with age provides a developmental extension of the common liability model (Vanyukov et al. 2012), indicating that adolescent use and dependence are more distinct from one another than adult use and dependence. Results for assessments ascertained in adulthood show a pattern more in line with what would be expected from the common liability model, whereby dependence symptom counts and simple questions about alcohol use provide similar measures of the latent construct from our factor models. Therefore, given the pattern and magnitude of factor loadings observed in this study, alcohol use frequency, alcohol use quantity, and alcohol dependence symptom count may have sufficiently similar measurement properties to enable clinical use of quantity and frequency questions in measuring alcohol dependence or problematic alcohol use, despite small, but significant differences. Indeed, a strength of the current study is that it is well powered to detect arguably minor differences in factor loadings.

The alcohol behavior latent factor was moderately heritable from ages 14 to 29 (ranging from 0.47 to 0.56), then declined at age 37 (*h* ^2^ = 0.24). These results are consistent with previous literature that has highlighted the common liability to externalizing disorders is heritable (Hicks et al. 2013), and that this common factor may become weaker with age (Vrieze et al. 2012). Given that dependence loads less strongly on this latent factor in adolescence, the heritability estimates in the present study indicate that genetic factors play a relatively larger role in observed relationship between alcohol use and dependence at younger ages. However, genetic influences on the latent factor dropped to 0.24 at the age 37 assessment, with large gains in non‐shared environmental factors, potentially highlighting that environmental influences are more important to similarity in these alcohol behaviors at older ages. This finding is contrary to the general trend finding of increasing heritability for alcohol behaviors as individuals age (i.e., McGue and Gottesman 2015). The discrepancy here may be due to the fact that previous twin study research has prioritized the individual heritability of these behaviors rather than examining their shared etiology (e.g., Deak et al. 2019; Geels et al. 2012; Jackson et al. 2002; Mbarek et al. 2015; Verhulst et al. 2015). Moreover, other research has indicated that heritability may not continue to increase or remain high with age, as a recent mega‐analysis on alcohol consumption found that heritability decreases after middle age (a^2^ at age 39 = 0.38 and 0.49 for males and females, respectively, but drops to 0.29 and 0.37 by age 70; Gustavson et al. 2025). Our findings presented in this study are also not entirely consistent with the high genetic correlations found between use and dependence in previous latent biometric and molecular genetic research (e.g., Dick et al. 2011; Grant et al. 2009; Kember et al. 2023; Kendler et al. 2010; Kranzler et al. 2019), though our results may shed light on differences seen between studies, given the developmental grading of genetic influences that were reported here. If considered at face value, our pattern of results at the age 37 assessment reflects a decline in the importance of genetic factors and an increase in the importance of nonshared environmental factors. Notably, however, estimated nonshared environmental effects include measurement error in twin models. The age 37 assessment may be less reliable than prior assessments because the age range was larger, and alcohol dependence symptoms were asked only over the past year (versus a longer recall period of 3–5 years at the other assessment ages). Previous research has indicated that reliability for alcohol dependence diagnoses was not significantly different between past‐year and lifetime recall periods (e.g., Hasin et al. 1997). Therefore, this variability in measurement estimates may be interpreted as measurement error, but it may also be indicative of a true increase in variance accounted for by the nonshared environment.

This study has its limitations. The study sample is a Minnesota birth cohort from birth years spanning the late 1970s to mid 1990s. While the sample is representative of children born in Minnesota in these years, it may have limited generalizability to other birth years or geographic regions. As a community sample, we cannot effectively evaluate measures of use and dependence among heavy users, or in populations where alcohol is scarcer or prohibited. At each age, we only had three measures: frequency, quantity, and dependence symptom count. Future work may consider individual alcohol dependence symptoms, other measures of use, and tests of external validity (e.g., of other forms of psychopathology or disease) to evaluate correlational patterns.

These results have both theoretical and practical implications for understanding alcohol behaviors as well as alcohol use disorder screening, assessment, and treatment. Clearly, and not surprisingly, alcohol use and dependence measures have different properties from adolescence to midlife, and their similarity to one another is not static over time. This study tested a factor structure with a single latent alcohol behavior factor to model the relationship between alcohol use frequency, quantity, and dependence. The latent factor here reflects a general tendency to engage with alcohol, similar to a general predisposition hypothesized in the common liability model. This study expands our understanding of this common liability by providing a developmentally graded understanding of the shared tendency to engage with these alcohol behaviors, and results indicate that the nature or measurement of this common liability changes with age. However, future research will be required to test its relationship to other characteristics such as illness or psychopathology. What can be inferred from the present findings is that alcohol use frequency, quantity, and dependence function similarly for individuals in their mid‐20s and later, but alcohol use frequency and quantity are more distinct from alcohol dependence symptom count at younger ages. In clinical application, screening questionnaires for alcohol use disorder that measure only frequency or quantity of use will function differently in adolescents than adults. Therefore, practitioners working with adults will continue to benefit from inclusion of simple and efficient questions of quantity and frequency of alcohol use. Additionally, the heritability for the shared liability for alcohol use and dependence decreases with age, while the effect of the nonshared environment increases. Interventions for decreasing alcohol consumption and/or AUD symptoms may benefit from assessing and changing environmental factors that drive alcohol use at midlife and beyond.

## Funding

This work was supported by the National Institute on Drug Abuse (Nos. R01 DA042755, R01 DA044283, R01 DA054087, and T32 DA050560) and National Institute on Alcohol Abuse and Alcoholism (No. R37 AA009367).

## Conflicts of Interest

The authors declare no conflicts of interest.

## Supporting information


**Table S1:** Descriptive statistics by cohort.
**Table S2:** Correlations among observed phenotypes.
**Table S3:** Association on recall period on alcohol dependence symptoms.
**Table S4:** Genetic correlations between latent factors.
**Table S5:** Genetic correlations between specific factors.

**Figure S1:** Primary results figure.

## Data Availability

The data that support the findings of this study are available on request from the corresponding author. The data are not publicly available due to privacy or ethical restrictions.
