# Supplementary material for: Etiological Development of Alcohol Use and Dependence From Adolescence to Midlife in a Longitudinal Community Study of Twins
Source: Alcohol Clin Exp Res (Hoboken). 2026 Apr 19;50:e70299. doi: 10.1111/acer.70299 (PMC13092882; doi:10.1111/acer.70299)
Supplement: Supplementary file 2 — Figure S1: Primary results figure. [file ACER-50-0-s002.docx]

**Supporting Information**

Etiological development of alcohol use and dependence from adolescence to midlife in a longitudinal community study of twins

Brooke A. Huizenga, MA^1^

Jordan D. Alexander, MA^1^

Robert F. Krueger, PhD^1^

Matt McGue, PhD^1^

Sylia Wilson, PhD^2^

Stephanie M. Zellers, PhD^3^

Scott I. Vrieze, PhD^1^

^1^Department of Psychology, University of Minnesota

^2^Institute of Child Development, University of Minnesota

^3^Institute for Molecular Medicine Finland, University of Helsinki

**Alcohol Use**

Responses for alcohol use frequency were ascertained ordinally. Response options for alcohol frequency included the following: 0=non-drinker, 1=less than once a month, 2=1-3 times a month, 3=1-2 times a week, 4=3-4 times a week, and 5=daily. Responses for alcohol use quantity were most commonly ascertained continuously. In some cases where participants were missing this data on quantity, data ascertained ordinally for a very similar question on a computerized substance use module at the same wave of assessment was used. These quantity data were thus harmonized by re-binning them into ordinal scale categories across wave. Response options for alcohol quantity included the following: 0=no use, 1=1-3 drinks per occasion, 2=4-6, 3=7-10, 4=11-20, 5=21-29, or 6=30+.

**Additional information on the sample**

The sample of twins consisted of three cohorts. The first two cohorts, the younger cohort and older cohort are from the Minnesota Twin Family Study (MTFS). MTFS is a community-representative longitudinal cohort study of monozygotic and same-sex dizygotic twins. Twins were assessed from age 11 for the younger cohort and age 17 for the older cohort into middle adulthood, with the most recent wave of assessment at target age 42. The third cohort is from the Enrichment Sample (ES). ES is also a longitudinal cohort study of monozygotic and same-sex dizygotic twins, where half of the cohort was oversampled for high risk of substance use disorders. They were also assessed from age 11 into adulthood, with the most recent wave of assessment at age 29. Note that only a small proportion of the ES cohort (~28.5%) were assessed at the age 21 assessment due to funding limitations. Additionally, they were not assessed at a standalone age 29 assessment, as the assessment conducted at this age occurred at the same time (with the same materials) as the midlife assessment conducted in the OC and YC cohorts, and thus the data collected at age 29 is included in the age 37 midlife assessment. See *Supporting Information Table 1* for a full breakdown of descriptive statistics stratified by wave of assessment and cohort.

**Effect of Recall Period**

Because recall period varied across waves of assessment, an analysis was conducted to determine whether recall period had a significant effect on number of symptoms reported for alcohol dependence symptom count. Analyses consisted of linear regression correcting for age and sex. Recall period for age 14 was past 3 years and recall period for age 17 was lifetime. Recall periods for ages 21, 24, and 29 were since last visit. Recall period for the age 37 assessment was past year. Results indicated that time since last visit was significantly associated with greater endorsement of alcohol dependence symptoms at every assessment wave other than age 37 (see *Supporting Information Table 3*). Because recall period was variable (i.e., dependent on time since last visit, and could vary based on whether an assessment was skipped by a participant) for assessments at ages 21, 24, and 29, primary analyses corrected for this. Ages 14 and 17 also show a significant association between dependence symptom endorsement and time since last visit (please note that there was also an age 11 wave of assessment that was omitted from all analyses due to low endorsement of substance use and dependence across the sample). However, primary analyses did not correct for recall period at ages 14 and 17, because the recall period was fixed for these assessment waves.

**Supporting Information Figures**

*Supporting Information Figure 1. Primary Results Figure*


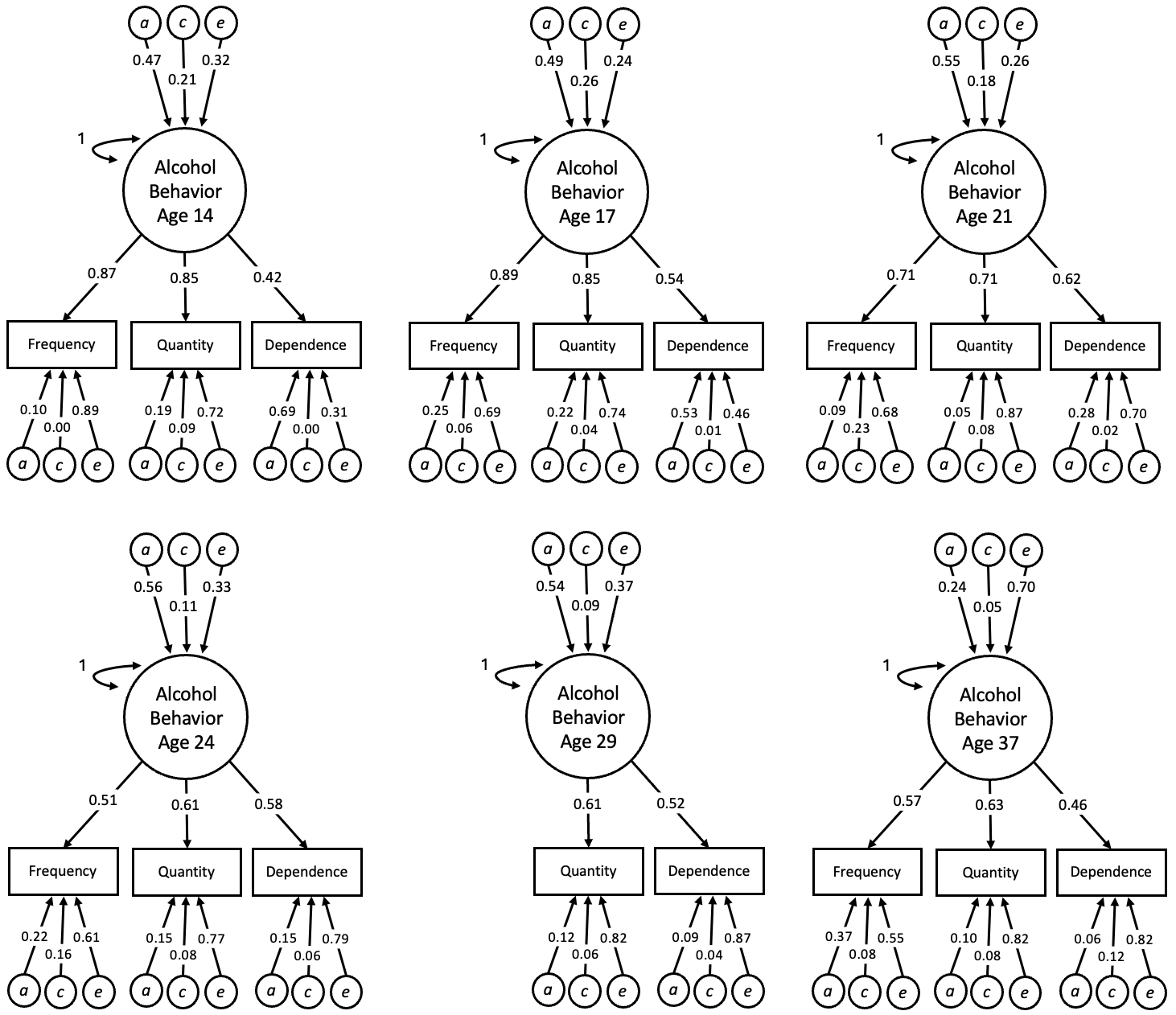


*Supporting Information Figure 1. Primary Results Figure*: This figure depicts both latent factor loadings and the ACE biometric decomposition for the latent and specific factor variance at all waves of assessment; for a full overview of the path model with residual correlations, please see *Figure 1* of the main text. Confidence intervals for standardized factor loadings and variance decomposition can be found in *Table 3* of the main text. Alcohol use frequency at age 29 is missing by design. Frequency = alcohol use frequency; Quantity = alcohol use quantity; Dependence = DSM-III-R Alcohol Abuse and Dependence combined symptom count; a = variance decomposition attributable to genetic effects; c = variance decomposition attributable to shared environmental effects; e = variance decomposition attributable to nonshared environmental effects.

**Overview of Supporting Information Tables**

*Supporting Information Table 1. Descriptive Statistics By Cohort:* Descriptive statistics for age, Frequency, Quantity, and Symptoms are shown as mean(SD); YC = younger cohort; OC = older cohort; ES = enrichment sample; MBD = missing by design; Frequency = alcohol use frequency; Quantity = alcohol use quantity; Dependence = DSM-III-R Alcohol Abuse and Dependence combined symptom count.

*Supporting Information Table 2. Correlations Among Observed Variables*: Alcohol use frequency at age 29 is missing by design. Frequency = alcohol use frequency; Quantity = alcohol use quantity; Dependence = DSM-III-R Alcohol Abuse and Dependence combined symptom count.

*Supporting Information Table 3. Association of Recall Period on Alcohol Dependence Symptoms*: * = significant at the 0.05 level; ** = significant at the 0.01 level; *** = significant at the 0.001 level. Regression slopes calculated as linear regressions and shown as estimate (standard error).

*Supporting Information Table 4. Genetic Correlations Between Latent Factors*: Correlations shown as correlation [95% CI]. Latent Factors are labeled by wave of assessment.

*Supporting Information Table 5. Genetic Correlations Between Specific Factors*: Correlations shown as correlation [95% CI]; Alcohol use frequency at age 29 is missing by design. Frequency = alcohol use frequency; Quantity = alcohol use quantity; Dependence = DSM-III-R Alcohol Abuse and Dependence combined symptom count.
